# Research hotspots and trends in the application of diffusion tensor imaging in ischemic stroke: a bibliometric analysis (2003–2024)

**DOI:** 10.3389/fneur.2025.1579598

**Published:** 2025-06-30

**Authors:** Xu Chen, Meifang Liu, Xiaolin Yang, Mengqi Yue, Haocheng Yu, Haiqiang Wang, Zirong Wang, Jing Shi, Yong Qiu

**Affiliations:** ^1^Second Clinical Medical College, Yunnan University of Chinese Medicine, Kunming, Yunnan, China; ^2^Yunnan Provincial Hospital of Traditional Chinese Medicine, The First Clinical Medical College of Yunnan University of Traditional Chinese Medicine, Kunming, China

**Keywords:** ischemic stroke, diffusion tensor imaging, bibliometric, function, biomarker

## Abstract

**Background:**

Ischemic stroke (IS) is a limited ischemic necrosis or softening of brain tissue caused by impaired blood supply to the brain, ischemia, and hypoxia, ultimately leading to various neurological dysfunctions. In recent years, with the continuous development of imaging technology, diffusion tensor imaging (DTI) has made many advances in the field of IS. However, bibliometric analysis in this field is still lacking. This study aims to investigate the present status of study progress, research hotspots, and possible research trends in the application of DTI in IS through bibliometric analysis methods.

**Methods:**

The Web of Science Core Collection (WoSCC) database was searched for relevant literature on DTI in IS from 2003 to 2024. Using VOSviewer, CiteSpace, and R package Bibliometrix to visualize and analyze countries, publications, authors, co-citations, and keywords.

**Results:**

A total of 493 papers from 45 countries were incorporated. The number of DTI-related publications in IS has been increasing year by year. The three primary publishing nations are the United States, China, and the United Kingdom. Harvard University is the research institution with the most extensive of publications. STROKE is the most contributing and cited journal in the field. Markus, Hugh S is the author with the highest number of publications. Thomalla, G. is the author with the highest number of citations. The analysis of keywords and co-cited literature can suggest the primary research directions and trends in the field containing diffusion tensor imaging, ischemic stroke, white matter (WM), Wallerian degeneration (WD), corticospinal tract (CST), fractional anisotropy (FA), recovery and biomarker for motor function.

**Conclusion:**

According to the bibliometric study's findings, this area is progressively gaining the attention of researchers and may become a research hotspot in IS. However, the current study still needs to strengthen cross-regional cooperation, and higher quality and broader research is still necessary to lay the foundation for reaching the consensus of various experts.

## 1 Introduction

Stroke ranks as the second foremost cause of mortality globally, accounting for 11.6% of all deaths, and is the third principal cause of the aggregate burden of death and disability, accounting for 5.7% of total disability-adjusted life years (1). The growing worry regarding neurological abnormalities post-stroke is particularly significant, as limb dysfunction, the most common sequela, can intensify the numerous hardships faced by patients and their families (2). Notwithstanding the progress in the management of acute IS and subsequent rehabilitation, the rate of post-stroke reliance persists at 20%– 30% (3). The escalating expense of healthcare has intensified the necessity for early and precise prediction of post-stroke outcomes to establish attainable rehabilitation objectives and alleviate the psychological and financial strain on patients and their families (4). The present evaluation of neurological abnormalities and rehabilitation remains inadequate and devoid of specialized instruments; thus, investigating a test for assessing motor function prognosis holds therapeutic significance.

DTI is a new imaging modality developed by Basser et al. (5) based on conventional MRI techniques. Diffusion is one of the most fundamental modes of motion of matter. While the other type of diffusion is direction-dependent and the distance of each molecule is not equal, which is called anisotropic, under the influence of no external environmental perturbations, in completely homogeneous water, each water molecule can perform a random Brownian motion, and the distance of its motion in each direction is equal, which is called isotropic (6, 7). In WM, due to myelin sheaths, water molecules diffuse faster along the fiber bundles but slower perpendicular to the fibers, leading to anisotropic diffusion (8, 9). DTI takes advantage of the fact that water molecules move anisotropically in the human body by using a Gaussian distribution model that describes molecules' three-dimensional (3D) displacement in terms of a 3 × 3 symmetric matrix. Correspondingly, each derived eigenvalue (λ_1_, λ_2_, or λ_3_; λ_1_ > λ_2_ > λ_3_) denotes the diffusivity in the direction of the corresponding eigenvector (*V*_1_, *V*_2_, or *V*_3_), and they are used to estimate scalar parameters, including the mean diffusivity [MD = (λ_1_ + λ_2_ + λ_3_)/3], the fractional anisotropy [FA = SQRT (0.5 × ((λ_1_ – λ_2_)^2^ + (λ_1_ – λ_3_)^2^ + (λ_2_ – λ_3_)^2^)/($\lambda_{1}^{2}$ + $\lambda_{2}^{2}$ + $\lambda_{3}^{2}$)], axial diffusivity (AD, λ_||_ = λ_1_) and radial diffusivity (RD, λ_⊥_ = (λ_2_ + λ_3_)/2). In addition, the number and volume of fibers in each pathway can be outlined by DTI fiber bundle tracer imaging (10). Since DTI may be parameterized to measure the extent of anisotropic diffusion of water molecules, it has broad use in several disorders, including IS (11), cerebral leukoencephalopathy (12), gliomas (13), Alzheimer's disease (14), Parkinson's disease (15), and amyotrophic lateral sclerosis (16), among others.

Bibliometric analysis, as a quantitative methodology, facilitates the examination of extensive and diverse literature, objectively and intuitively illustrating historical academic research endeavors and outcomes while aiding in minimizing the impact of human-induced bias in paper evaluation (17). bibliometric analysis is now widely used in medical and healthcare research (18). A review of DTI as a biomarker for predicting exercise outcomes in action-induced ischemia has been published. However, there is an absence of related bibliometric analyses to examine the current state of research further, more thoroughly and impartially. Thus, we employed three analytical software tools, VOSviewer, CiteSpace, and Bibliometrix, to construct knowledge maps to analyze relevant information, research hotspots, and prospective research trends in the subject.

### 1.1 Search strategy

We systematically searched the Web of Science Core Collection (WoSCC) database (https://webofscience.clarivate.cn/wos/woscc/advanced-search). The searched terms were obtained from the MeSH (https://www.ncbi.nlm.nih.gov/mesh). The search formula is as follows: #1=((((((((TS=(Imaging, Diffusion Tensor)) OR TS=(Diffusion Tensor Magnetic Resonance Imaging)) OR TS=(Diffusion Tensor MRI)) OR TS=(Diffusion Tensor MRIs)) OR TS=(MRI, Diffusion Tensor)) OR TS=(DTI MRI)) OR TS=(Diffusion Tractography)) OR TS=(Tractography, Diffusion)) OR TS=(DTI), #2= (((((((((((((((((((((((TS=(Ischemic Strokes)) OR TS=(Stroke, Ischemic)) OR TS=(Ischaemic Stroke)) OR TS=(Ischaemic Strokes)) OR TS=(Stroke, Ischaemic)) OR TS=(Cryptogenic Ischemic Stroke)) OR TS=(Cryptogenic Ischemic Strokes)) OR TS=(Ischemic Stroke, Cryptogenic)) OR TS=(Stroke, Cryptogenic Ischemic)) OR TS=(Cryptogenic Stroke)) OR TS=(Cryptogenic Strokes)) OR TS=(Stroke, Cryptogenic)) OR TS=(Cryptogenic Embolism Stroke)) OR TS=(Cryptogenic Embolism Strokes)) OR TS=(Embolism Stroke, Cryptogenic)) OR TS=(Stroke, Cryptogenic Embolism)) OR TS=(Wake-up Stroke)) OR TS=(Stroke, Wake-up)) OR TS=(Wake up Stroke)) OR TS=(Wake-up Strokes)) OR TS=(Acute Ischemic Stroke)) OR TS=(Acute Ischemic Strokes)) OR TS=(Ischemic Stroke, Acute)) OR TS=(Stroke, Acute Ischemic). The final expression is #1 AND #2, with publication year from January 1, 2003, to December 31, 2024.

### 1.2 Literature inclusion and exclusion criteria

Inclusion criteria: (1) Literature source: WoSCC; (2) Time frame: articles published between 2003 and 2024; (3) Document types: article and review article; Exclusion criteria: (1) irrelevant to the topic; (2) proceeding paper, meeting abstract, editorial material, and early access, letter; (3) retracted or overlapping studies.

### 1.3 Search result

**Figure 1:**
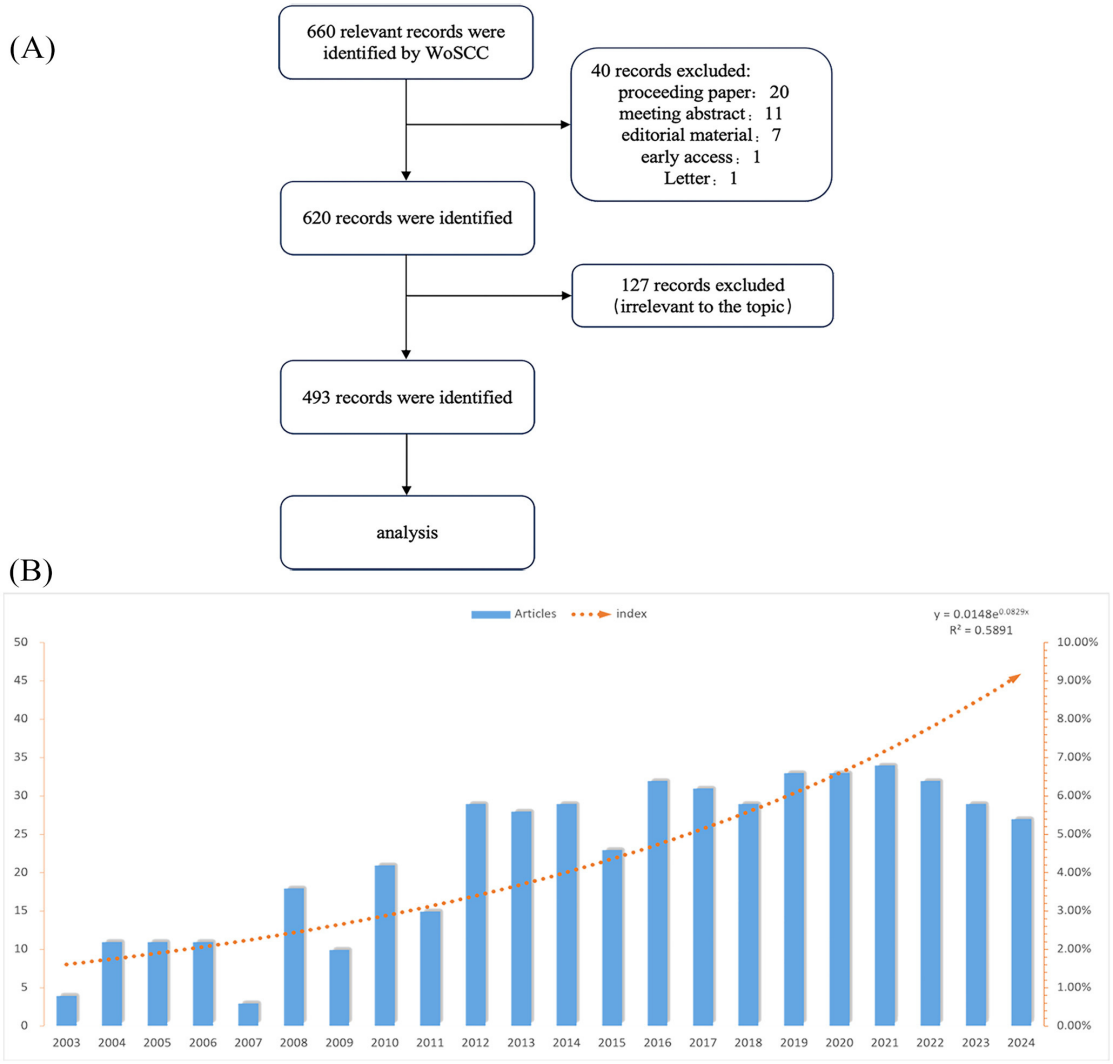
**(A)** Publications screening flowchart. **(B)** Annual publications in the field of the application of DTI in IS.

Two independent researchers reviewed each paper's title and abstract to filter the papers further. After excluding 40 records (proceeding paper: 20, meeting abstract: 11, editorial material: 7, early access: 1, and letter: 1) + 127 papers that did not meet the inclusion criteria, a final set of 493 papers was obtained (Figure 1).

### 1.4 Data extraction and analysis

The data was exported in the form of a plain text file, full record, and cited references. VOSviewer (version 1.6.20) is a bibliometric analysis software that extracts essential information from many publications and is frequently utilized to construct collaboration, co-citation, and co-occurrence networks. Our study utilized software to depict countries/regions, institutions, journal and authorship collaborations, and coupling mapping. In the VOSviewer-generated map, a node signifies an entity such as a country, institution, journal, or author. The size and color of the nodes represent the quantity and classification of these things, respectively. The thickness of the lines connecting nodes indicates the extent of collaboration or co-citation among the items.

CiteSpace (version 6.3.R1) is a software developed by Professor Chen C for bibliometric analysis and display. We employed CiteSpace to delineate the dual-map overlay of journals and to examine references utilizing Citation Bursts.

Bibliometrix (https://www.bibliometrix.org) is a R package designed for bibliometric analysis. It offers a range of functions for data extraction, processing, and visualization within the R environment. Bibliometrix was applied to provide a graphical representation of authors, cited references, and thematic maps.

## 2 Result

### 2.1 Analysis of publication

Based on the search strategy, we incorporated a total of 493 articles. Figure 1B illustrates that the trend line indicates a consistent increase in the number of articles published from 2003 to 2024, suggesting a growing interest in applying tensor imaging within the domain of IS. The bar chart can be distinctly categorized into two phases, with 2012 serving as the turning point. Prior to 2012, the publication of articles was limited, totaling 104 articles, which constituted 21.10% and averaged 11.56 articles annually. Conversely, post-2012, the volume of published articles significantly increased, totaling 389 articles, representing 78.90% and averaging 29.92 articles per year. This indicates that over the past 13 years, research on IS in conjunction with tensor imaging has sustained a high level of interest.

### 2.2 Analysis of countries/regions

**Figure 2:**
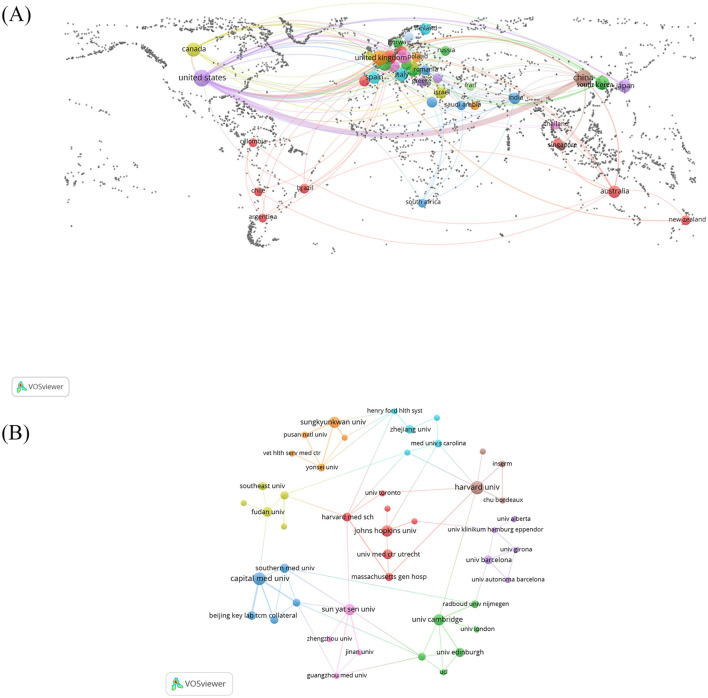
**(A)** The geographical distribution on research of the application of DTI in IS. **(B)** The visualization of institutions.

Publications in this domain predominantly originate from 45 countries. The Table 1 enumerates the top 10 countries by publication count. Notably, three countries exceed 50 publications: the United States (*n* = 144), China (*n* = 128), and the United Kingdom (*n* = 55). Collectively, these three nations represent 49.85% of the total publication volume. Regarding international cooperation, we utilize VOSviewer to generate national geo-visualization maps (Figure 2A). The minimum document requirement for a country was established at 3, with 23 out of 45 countries satisfying this criteria. This geo-visualization atlas depicts each node as a country, with connecting lines illustrating the cooperation across countries. The figure demonstrates that the issuing countries are predominantly located in Europe, with Asia and North America following. The most pronounced line of cooperation between the United States and China signifies their closest collaboration, while the most intersecting lines with other nations indicate the United States' extensive collaboration with those countries.

**Table 1:** Top 10 countries and institutions in the field of the application of DTI in IS.

**Rank**	**Country**	**Documents**	**Institution**	**Documents**	**Country**
1	United States	144	Harvard University	19	United States
2	China	128	Capital Medical University	17	China
3	United Kingdom	55	Sun Yat-sen University	14	China
4	Germany	41	University of Cambridge	14	United Kingdom
5	South Korea	38	Johns Hopkins University	13	United States
6	Canada	32	Sungkyunkwan University	13	South Korea
7	France	32	University of Calgary	12	Canada
8	Netherlands	30	Fudan University	10	China
9	Japan	23	University Medical Center Utrecht	10	Netherlands
10	Italy	20	Southern Medical University	9	China

### 2.3 Analysis of institution

The Table 1 illustrates the top 10 institutions, of which nine have exceeded 10 publications: Harvard University (*n* = 19), Capital Medical University (*n* = 17), Sun Yat-sen University (*n* = 14), Cambridge University (*n* = 14), Johns Hopkins University (*n* = 13), Sungkyunkwan University (*n* = 13), University of Calgary (*n* = 12), Fudan University (*n* = 10) and University Medical Center Utrecht (*n* = 10). We utilized VOSviewer to draw a relationship map predicated on a minimum publishing threshold of four or more (Figure 2B). Harvard University and Harvard Medical School engage in the most collaborations with other universities (*n* = 8), followed by Sun Yat-sen University (*n* = 7), signifying that these institutions occupy a key position in this field.

### 2.4 Analysis of journals and co-cited journals

**Figure 3:**
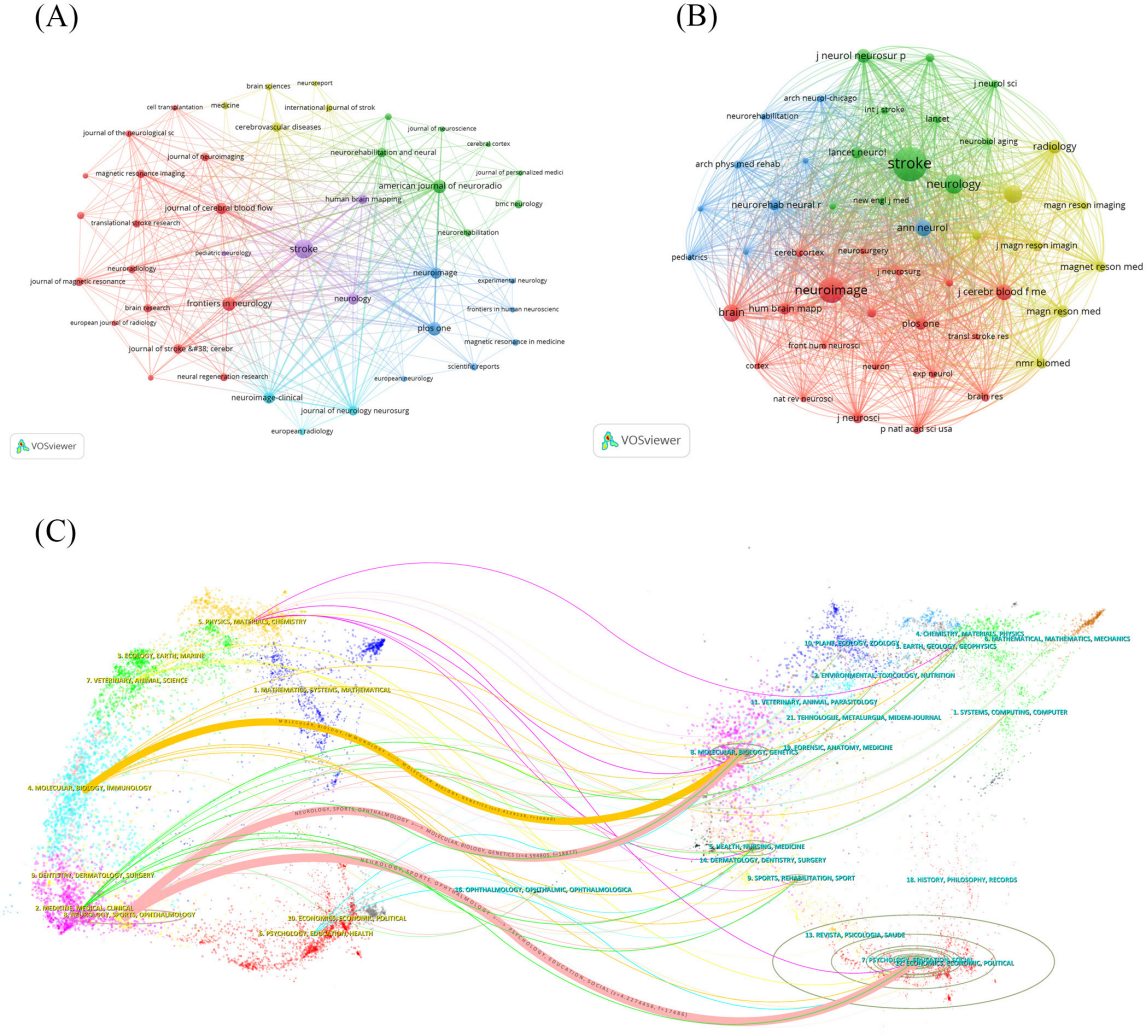
Journals analysis. **(A)** The visualization of journals. **(B)** The visualization of co-cited journals. **(C)** The dual-map overlay of journals.

A total of 493 articles were published across 187 journals in this domain. As illustrated in Table 2, we identified the top 10 journals based on publication number. The journal with the highest publication count was Stroke (*n* = 33, IF = 7.8), followed by the Frontiers in Neurology (*n* = 19, IF = 2.7) and the American Journal of Neuroradiology (*n* = 17, IF = 3.1). Neurology (*n* = 13, IF = 8.4) possessed the highest impact factor (IF). We selected 45 journals for the journal network visualization using VOSviewer software, stipulating a minimum publishing threshold of three or more (Figure 3A). The image reveals that Stroke, possessing the highest publication count, exhibits the most intricate network of relationships, signifying the most significant degree of collaboration with other journals.

**Table 2:** Top 10 journals and co-cited journals in the field of the application of DTI in IS.

**Rank**	**Journal**	**Counts**	**IF**	**Region**	**Co-cited journal**	**Counts**	**IF**	**Region**
1	Stroke	33	7.9	USA	Stroke	2,656	7.9	USA
2	Frontiers in Neurology	19	2.7	Switzerland	NeuroImage	1,535	4.7	USA
3	American Journal of Neuroradiology	17	3.1	USA	Neurology	902	8.4	USA
4	PLOS ONE	16	2.9	USA	American Journal of Neuroradiology	744	3.1	USA
5	Neurology	13	8.4	USA	Magnetic Resonance in Medicine	668	3.0	USA
6	Journal of Cerebral Blood Flow and Metabolism	12	4.9	USA	Brain	670	11.9	England
7	NeuroImage	12	4.7	USA	Journal of Cerebral Blood Flow and Metabolism	578	4.9	USA
8	Neuroimage-Clinical	12	3.4	Netherlands	Annals of Neurology	568	8.1	USA
9	Cerebrovascular Diseases	8	2.2	USA	Journal of Neurology, Neurosurgery and Psychiatry	434	8.8	England
10	Human Brain Mapping	8	3.5	USA	Radiology	427	12.1	USA

Cited journals can reflect the extent to which a specific journal contributes to study within the discipline. Among the 2,351 referenced publications, 47 were cited over 80 times. The Table 2 presents the top 10 most cited journals, with Stroke (*n* = 2,656, IF = 7.9) and Neuroimage (*n* = 1,535, IF = 4.7) quoted more than 1,000 times. The most significant IF for cited references was published in Radiology (*n* = 427, IF = 12.1). We utilized VOSviewer software to create a relationship network map of the referenced journals, as illustrated in the Figure 3B. This network is intricate and demonstrates that each cited journal is tightly interconnected with others, particularly Stroke.

In conclusion, two significant points exist: 11 of the 15 journals depicted in the figures originate from the United States, indicating more emphasis on study in this domain inside the United States compared to other nations. The analysis of the citing and cited journals implies that Stroke occupies a highly significant role in this field of study.

Based on the relationship between the citing and cited journals, we plotted a double map overlay of the journal (Figure 3C). The left side illustrates the clustering of citing journals, while the right side depicts the clustering of cited journals. The interconnected lines in the center represent the citation relationships, with the thickness of the lines indicating the density, namely citation intensity. The most conspicuous elements include two pink lines: one representing the fields of Neurology, Sports, and Ophthalmology from Molecular Biology and Genetics and another from Psychology, Education, and Social fields. Additionally, one yellow line represents the fields of Molecular Biology and Immunology from Molecular Biology and Genetics. The arrangement of these lines indicates the presence of both aggregated and decentralized research kinds, suggesting interdisciplinary convergence and predicting extensive opportunities for the advancement of this sector.

### 2.5 Analysis of authors and cited authors

**Figure 4:**
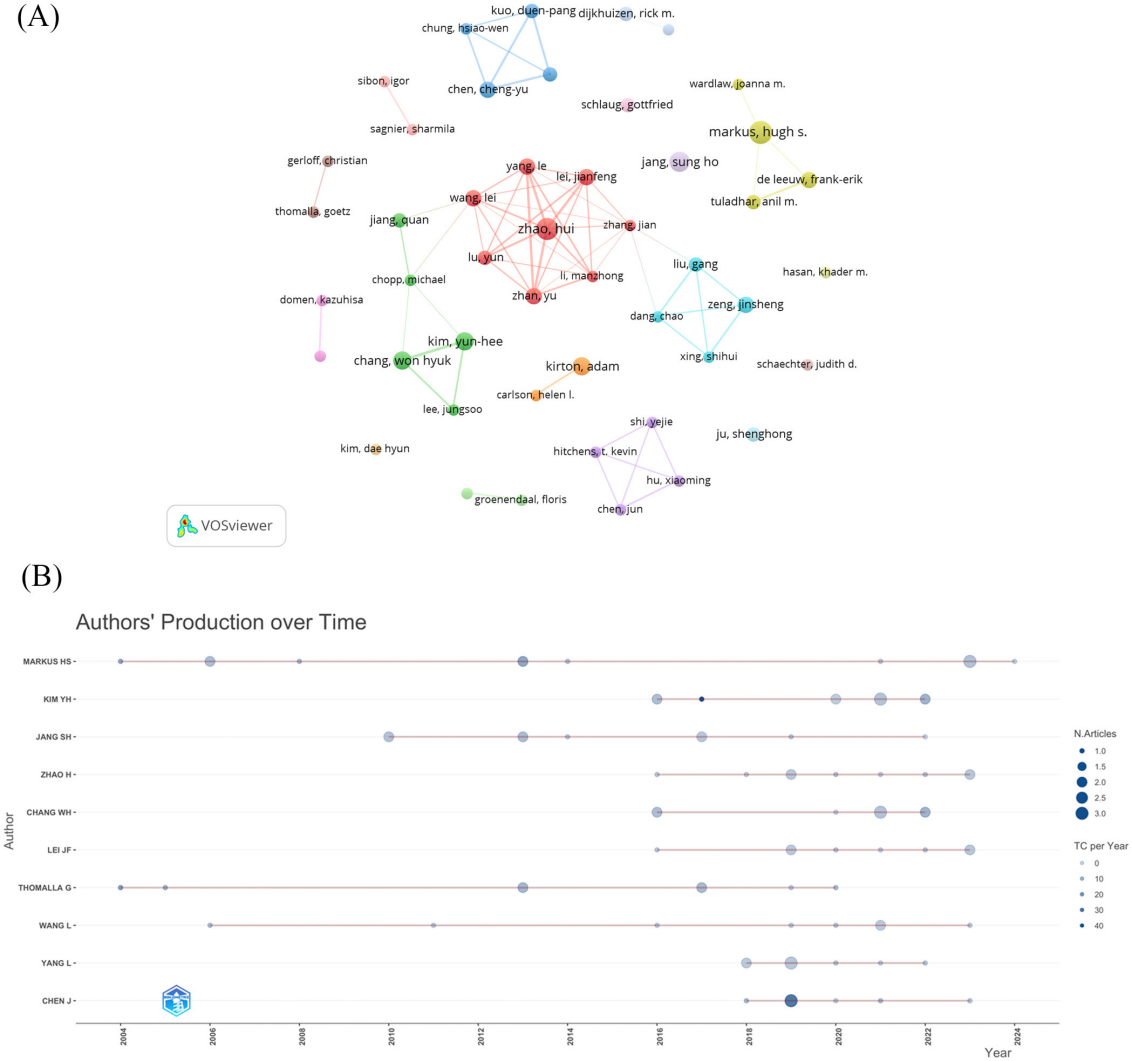
Authors analysis. **(A)** The visualization of author's collaboration. **(B)** Author's production over time.

Of these 493 documents, 3,045 authors contributed to these 493 papers. We identified the top 10 authors based on previous posting volume (Table 3), with the leading four being Markus, Hugh S (*n* = 12), Zhao, Hui (*n* = 9), Jang, Sungho (*n* = 9), and Kim, Yun-Hee (*n* = 9). We adopted VOSviewer software to construct a mapping of the collaborative network among 47 writers, stipulating a minimum publication threshold of four (Figure 4A). As shown in the figure, it can be roughly divided into four larger collaborative networks, and we find that the largest collaborative networks in the middle are from 3 countries: China, the United States, and South Korea, where the United States serves as an intermediate bridge connecting with authors from South Korea and China. The other three collaborative networks are from the UK, USA, and China, respectively. The other authors lack intimate collaboration with one another. The top 10 co-authors listed all have more than 100 citations, with the highest being Thomalla, G (*n* = 170), followed by Glauche, V, Röther, J, and Weiller, C, all with 129 citations (Table 3).

**Table 3:** Top 10 authors and cited authors in the field of the application of DTI in IS.

**Rank**	**Authors**	**Articles**	**Cited author**	**Local citations**
1	Markus, Hugh S	12	Thomalla, G	170
2	Zhao, Hui	9	Glauche, V	132
3	Jang, Sungho	9	Röther, J	132
4	Kim, Yun-Hee	9	Weiller, C	132
5	Chang, Wonhyuk	8	Beaulieu, C	131
6	Lei, Jianfeng	8	Blasco, G	109
7	Thomalla, Götz	8	Castellanos, M	109
8	Kirton, Adam	7	Daunis-i-estadella, J	109
9	Wardlaw, Joanna m	7	Pedraza, S	109
10	Chen, Cheng-yu	6	Puig, J	109

Subsequently, we employed Bibliometrix to generate a timeline graph illustrating the authors' articles' annual publication (Figure 4B). The graph indicates that MARKUS HS from Cambridge University consistently published between 2004 and 2024, with four contributions in the last 2 years. In contrast, the other authors lack a sufficient publication history or have published fewer articles in recent years, suggesting that MARKUS HS is the most promising prospective collaborator in this field.

### 2.6 Analysis of keywords

**Figure 5:**
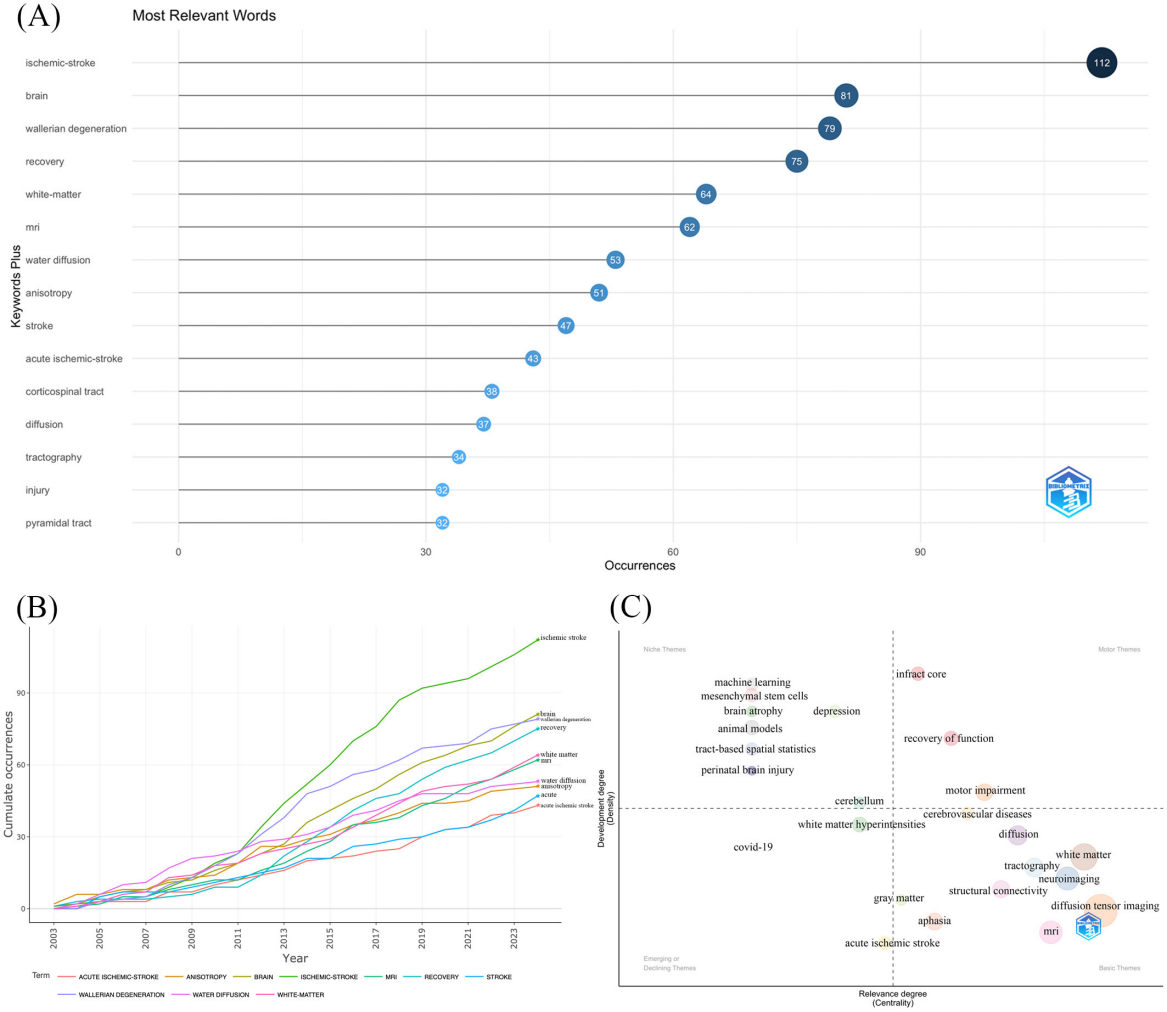
Keywords analysis. **(A)** Top 15 relevant keywords. **(B)** Words' frequency over time. **(C)** Thematic map of keywords.

We utilized Bibliometrix to examine the 15 most frequently occurring author keywords (Figure 5A). The top five occurrences were ischemic stroke (*n* = 112), brain (*n* = 81), Wallerian degeneration (WD) (*n* = 79), recovery (*n* = 75), white matter (*n* = 64), and MRI (*n* = 62). Furthermore, we also constructed the Words' frequency over time map (Figure 5B) and the Thematic Map (Figure 5C) to enhance the analysis of research hotspots and trends derived from the keywords.

We analyzed word frequency over time for the keywords with the most pronounced variations in word frequency (Figure 5B). The figure illustrates the analysis of word frequency changes for the five keywords exhibiting the most significant fluctuations: ischemic stroke, Wallerian degeneration, brain, recovery, and white matter. The frequency of these keywords has consistently risen each year, particularly following 2011. The escalation is intensifying, indicating that these five keywords may represent the research trend in this domain.

Thematic map (Figure 5B) is where the vertical axis represents density, and the horizontal axis represents centrality. Density quantifies the cohesion among nodes, while centrality reflects the extent of association among various themes. These two criteria assess the degree of development and significance of specific issues. In a thematic network, a node's centrality and significance increase with the number of relationships it has with other nodes, rendering it more pivotal within the network. Likewise, the cohesiveness among nodes signifies the density of a research domain, illustrating its capacity for development and sustainability. The thematic map depicted in the figure is segmented into four quadrants. The upper right quadrant exhibits both high density and high centrality, indicating that its content is significant and possesses growth potential. Consequently, we can conjecture that the terms infarct core, recovery of function, and motor impairment are significant and possess developing potential in this research domain.

### 2.7 Analysis of reference

**Figure 6:**
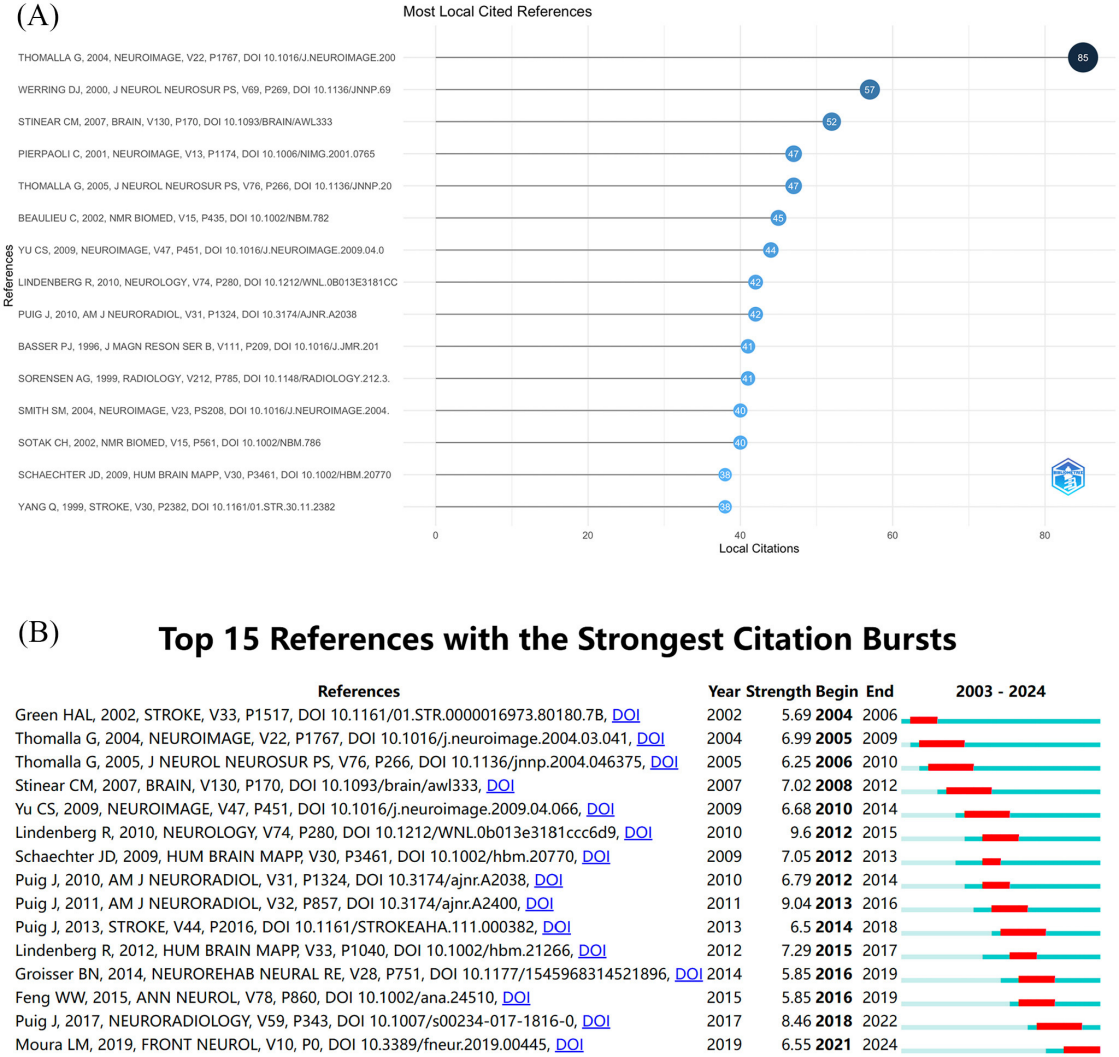
References analysis. **(A)** Top 15 cited references. **(B)** Top 15 references with the strongest bursts.

The compilation of reference citations in publications can be considered a repository of scientific knowledge (17). The Figure 6A numerates the top 15 cited publications, all with >38 citations, and the top 3 with more than 50 citations, which are “Diffusion tensor imaging detects early Wallerian of the pyramidal tract after ischemic stroke” (NEUROIMAGE, IF = 4.7) by Götz Thomalla, and his team (19), followed by “Diffusion tensor imaging can detect and quantify corticospinal tract degeneration after stroke” (Journal of Neurology, Neurosurgery and Psychiatry, IF = 8.8) by David J Werring and his team (20), followed by Cathy M. Stinear and his team with “Functional potential in chronic stroke patients depends on corticospinal tract integrity” (BRAIN, IF = 11.9) (21). The 15 referenced papers comprised 12 experimental studies and 3 reviews. Notably, all articles discussed fiber tracts, with 8 specifically mentioning the pyramidal tract or CST and 11 discussing FA. This signifies that scholars in this field are prioritizing discussions on CST and anisotropy. Other topics included WD (19, 22–25), apparent diffusion coefficient (ADC) (11, 26, 27), fiber tract integrity (20, 28), the net effect of degeneration and remodeling of bilateral CST (29), intervoxel measures of structural similarity (30), asymmetry indices and fiber number (31), apparent diffusion anisotropy, and potential marker (27) among others. This discussion centers on three factors that alter the brain microstructure following cerebral infarction: techniques of diagnosis and prognosis.

The most vigorous citation burst shows how the citation strength of the cited literature has changed over time. The Figure 6B displays the top 15 papers from the initiation to the cutoff period, with two papers exhibiting the highest citation intensity in the latest 3 years: “Diffusion Tensor Imaging as a Prognostic Biomarker for Motor Recovery and Rehabilitation after Stroke” and “Diffusion Tensor Imaging Biomarkers to Predict Motor Outcomes in Stroke: A Narrative Review.” Both publications cover biomarkers for motor recovery after stroke, potentially indicating a contemporary research trend.

## 3 Discussion

### 3.1 General information

Of the 493 publications analyzed in this study were published 104 (21.10%) prior to 2012 and 389 (78.90%) subsequent to 2012, indicating that research on this topic received insufficient attention before 2012 while garnering significant interest from researchers thereafter. The primary issuing nations are the United States, China, and the United Kingdom, collectively representing over fifty percent of the total articles, with the United States and China exhibiting the most significant relationship. The leading 10 issuing organizations are predominantly located in China and the United States, with 9 entities producing over 10 articles each. The predominant partnerships with other institutions are with Harvard University and Sun Yat-sen University. Among the 187 journals, STROKE has the most enormous number of publications, totaling 33. The leading 10 journals with the most publications possess an IF below 10, signifying a need for enhancement in the quality of articles within this study domain. There are 2,351 cited journals, with 47 exhibiting a citation frequency exceeding 80; among them, two journals, STROKE and NEUROIMAGE, both have a citation frequency surpassing 1,000, which shows that these two journals have contributed the most to this research ground. The network diagrams illustrating the relationships between journals and their reference counterparts are intricate, indicating a strong interconnection across journals. Two significant points merit attention: (1) STROKE, a journal, exhibits the most complicated connections among both citing and cited journals, signifying its paramount importance in this research realm; (2) 11 of the 15 journals depicted in the figures originate from the United States, suggesting a greater emphasis on research in this field within the United States compared to other nations. The author with the most posts is Markus, Hugh S from the University of Cambridge in the UK, who is also the most promising prospective collaborator for the future. The most significant network of partnerships is made up of three countries: China, the United States, and South Korea, with other larger networks coming from the United Kingdom, the United States, and China. Authors from other regions have not yet established collaborative links with one another.

### 3.2 Research hotspots and trends

Analyzing the keywords extracted from the publications included in this study can directly extract the research hotspots and research frontiers in this field. Likewise, analyzing the references contained in the literature can also indirectly suggest the research hotspots and research frontiers in this field. Since they are complementary to one another, combining the two can make a comprehensive analysis more convincing. Therefore, we made Words' frequency over time map and Thematic Map to analyze the keywords. The predominant five keywords are ischemic stroke, brain, Wallerian degeneration, recovery, white matter, and MRI, signaling that these terms are probable focal points in this research domain. The most notable temporal increases in frequency pertain to ischemic stroke, Wallerian degeneration, brain, recovery, and white matter, all of which exhibit a rising prevalence throughout the years, indicating that these concepts may be crucial to ongoing research in this domain. The keywords occupying the upper right quadrant of the Thematic Map are infarct core, recovery of function, and motor impairment, indicating that these phrases are significant and potentially advantageous in this domain. Subsequently, we examined the 15 most frequently cited references in the selected articles and determined that the predominant topic was fiber tract and anisotropy, with 8 articles specifically referencing the pyramidal tract or CST. This was followed by discussions on WD, ADC, apparent diffusion anisotropy, fiber tract integrity, the net effects of degeneration and remodeling of bilateral, intervoxel measures of structural similarity, asymmetry indices, and fiber count, as well as potential markers. This indicates that these subjects are focal points for researchers and may represent potential research hotspots and trends. Ultimately, we examined the variation in citation intensity of referenced documents over time and discovered that the two documents with the highest citation intensity in the latest three were both reviews, covering biomarkers for motor recovery after stroke, which suggests that this subject is currently a primary focus for researchers in latest years and may represent a prospective research trend in the future. In conclusion, we consolidated the terms that were identical or analogous across various methodologies, eliminated specific extraneous terms, and ultimately presented the following findings: (1) The research hotspots in this research area are most likely to focus on the following keywords: diffusion tensor imaging, ischemic stroke, white matter, corticospinal tract, Wallerian degeneration, fractional anisotropy; (2) Future research trends are likely to be centered around the following keywords: recovery, biomarker for motor function; (3) Some of the topics of discussion that should not be disregarded: #1 DTI using the combination of parameters; #2 net effect of degeneration and remodeling of bilateral CST; #3 methods of determining the extent of functional recovery.

WM, according to the study (1) (32), the volume of WM is 456 ± 48 cm^3^ in men and 392 ± 42 cm^3^ in women, which accounts for ~40% of total human brain volume. WM tracts can be divided into three major categories according to their connectivity and functionality: (1) projection fibers—ascending and descending tracts connecting parts of the cerebral cortex and subcortical structures such as deep gray nuclei, the brainstem, cerebellum, and spinal cord—include pyramidal tracts, thalamic radiations and optic radiations, (2) association fibers—connections between various cortical subregions within the ipsilateral hemisphere—can be long or short and include subcortical U fibers, the cingulum, superior and inferior longitudinal fasciculi, the occipitofrontal fasciculus, and the uncinate fasciculus, (3) commissural fibers—the link connecting homologous areas of the bilateral hemispheres—include the corpus callosum and the anterior commissure (3) (33). Microscopically, WM consists of axons, oligodendrocytes, and astrocytes. Axons are the largest element of WM by volume and occupy about 87% of its space (4) (34). WM has lower blood flow than gray matter (GM), and there is little collateral circulation, especially in deep WM (2) (35). However, WM is heavily dependent on a continuous supply of oxygen and glucose, and a series of damaging changes occur in WM after IS. Some animal studies (36) have shown that excessive glutamate release, activation of ATP receptors, oxidative stress, inflammation, and apoptosis all lead to oligodendrocyte death and axonal damage after ischemic brain injury. A significant portion of the clinical trial research has focused on the use of MRI techniques for the non-damaging detection of cerebral WM, particularly DTI.

DTI is an MRI technique based on quantifying the random diffusion of water molecules (37), which is vital in elucidating the extent and potential effects of microstructural damage to the cerebral WM and is superior to conventional MRI. Hence, the majority of researchers in this domain have focused on examining cerebral WM structure by DTI techniques, and most have discovered that the integrity of cerebral WM correlates with functional recovery following IS. Etherton et al. (38) showed that WM structural integrity is associated with poor early neurological outcomes independent of ischemic tissue outcomes. A study (39) comparing the severity of symptomatic deficits 24–72 h and 3 months after cerebral infarction found that the integrity of the hemispheric WM decreased significantly with time, whereas patients with fewer missing distal WM fiber bundles recovered motor function better. Ingo et al. (40) found that the FA value of normal-appearing white matter (NAWM) was significantly lower in the acute IS group than in the control group and was significantly positively correlated with the motor function of patients, suggesting that the microstructural integrity of the NAWM better reflects the severity of motor impairment in stroke patients. Etherton et al. (41) discovered that diminished FA values in the NAWM contralateral to the acute IS lesion were independently correlated with the mRS after 90 days, revealing that cerebral WM integrity may influence functional recovery post-stroke. Sagnier et al. (42) analyzed the data from the DTI test within 72 h of the beginning of acute IS and the findings of the mRS follow-up at 1 year after stroke, discovering a strong association between the FA value of the NAWM and motor function. The FA values of the NAWM predicted mRS at 1-year post-stroke onset (β = −0.24, *P* = 0.04), indicating that early DTI parameters of the NAWM serve as independent predictors of functional outcomes and may provide supplementary markers in recovery studies following stroke. We observed the application of DTI in WM detection after IS primarily relates to the integrity of WM and functional recovery after IS. Researchers concentrate a lot of emphasis on this element, which has also been a hot topic of study recently.

The CST is the downstream projection tract connecting motor areas and the spinal cord (33). It mainly originates from the pyramidal cells of the cortex in the upper and middle precentral gyrus and the anterior part of the paracentral lobule of the brain and then passes down through the internal capsule and the peduncles of the brain to form left-right cross-traversal structures at the cones of the medulla oblongata, which are WM nerve fibers in charge of limb movement functions (43, 44). When IS occurs, WD changes occur in the CST after ischemia and hypoxia (24). Waller (45) first proposed WD in 1,850 when they observed changes in damaged distal axons during unilateral excision of the glossopharyngeal nerve at the level of the frog's larynx. WD is a process characterized by the progressive disintegration of distal axons, accompanied by demyelination, following damage to the neuronal cell body or proximal axon. This phenomenon can occur in both the peripheral nervous system and central nervous system, with central nervous system WD predominantly affecting the CST, where cerebral infarction is a primary causative factor (46). Researchers' studies on WD in the central nervous system have primarily focused on the CST, with a few studies on other sites, such as Pinter et al. (39) on WD in the corpus callosum, Liu et al. (47) on WD in the medial dome of the hippocampus after hippocampal resection, and Ingo et al. (40) on the cingulate gyrus. In 1989, Kuhn et al. (48) described four stages of WD changes, and this study focused on the CST: the first stage occurs about 3–4 weeks after injury and shows axonal degeneration with minor biochemical changes; the second stage occurs 4–10 weeks after injury and is characterized by the disintegration of myelin proteins but an increase in the hydrophilicity of the degenerated tissues with the lipids inside the myelin sheaths intact; the third stage occurs at 10–14 weeks after injury, when the hydrophilicity of the degenerated tissue increases significantly, with myelin fat destruction and gliosis; In the fourth stage, selective neuronal necrosis occurs in the affected area several months to 1 year after the onset of the injury, resulting in atrophy of the corresponding area visible to the naked eye. When WD occurs in the CST due to ischemia and hypoxia, the degree of anisotropic diffusion of water molecules in the damaged CST changes due to structural changes. Although conventional MRI can show WD changes, it has low sensitivity for early WD, and it is difficult to quantify the severity of WD (49). Advancements in imaging techniques have enabled researchers to utilize DTI to identify the diffusion characteristics of water molecules and detect WD at an early stage, demonstrating great sensitivity and the ability to quantify WD (19). Therefore, researchers have since adopted DTI imaging for WD studies. The commonly used parameters of DTI are FA, MD, RD, and AD. FA is the most commonly used parameter in DTI, and FA refers to the proportion of changes in the diffusion tensor component caused by anisotropy changes throughout the diffusion tensor process (50). The value of FA ranges from 0 to 1. The higher the anisotropy fraction in the WM region of the brain, the faster water molecules are diffused along the direction of the WM fibers (51), which is calculated by the formula:


$$\begin{array}{l} FA\;=\;\sqrt{\frac{3}{2}}\frac{\sqrt{\;{\left(\;\lambda_{1}-\lambda_{2}\right)}^{2}\;+\;{\left(\;\lambda_{1}-\lambda_{3}\right)}^{2}\;+\;{\left(\;\lambda_{2}-\lambda_{3}\right)}^{2}\;}}{\sqrt{\lambda_{1}^{2}+\;\lambda_{2}^{2}\;+\;\lambda_{3}^{2}\;}} \end{array}$$

λ_1_, λ_2_, and λ_3_ are eigenvalues that define the diffusion size. MD denotes the diffusion rate of water molecules per unit time in mm^2^/s (52), typically represented as the ADC and the average diffusion coefficient. MD can be used to discriminate between cerebrospinal fluid and brain tissue (53), and this index can provide a clear and good comparison between white and gray matter during DTI analysis (54). RD and AD represent the integrity of myelin sheaths of nerve fibers and axons, respectively (55). FA is the most often utilized parameter in clinical practice, probably due to the substantial difference between gray matter and WM, resulting in more accurate FA measurements. Additionally, FA represents a physical property of the tissue that remains invariant with coordinate system rotation. Moreover, values derived from the same specimen can be compared across different time points, imaging modalities, and subjects (56). Yang et al. (27) found that ADC values and FA of acute stroke patients would change differently over time in three main ways: (1) ADC decreased, and FA increased; (2) ADC and FA both decreased; (3) ADC increased, and FA decreased. The authors hypothesized that the level of FA could reflect the degree of cell swelling and cell membrane degradation, i.e., the severity of ischemic injury. Møller et al. (57) found that a decrease in FA reflected the disruption of the integrity of the axon, the onset of WD, and the more pronounced the decrease in FA, the more severe the degree of WD. In summary, academics have investigated the occurrence and quantitative indicators of WD from the standpoint of structural alterations following CST injury, and they have discovered that FA is the most advantageous sign. This prompted the FA a hot topic in this field.

A variety of symptoms may manifest following an IS, encompassing motor, sensory, speech, swallowing, cognitive, psychological, and other impairments. In the field of DTI application for IS, certain researchers have focused on the restoration of cognitive function (58) and the recovery of aphasia (59). Nevertheless, the predominant focus among researchers has been on the rehabilitation of limb motor function. This emphasis may be attributed to two primary factors: (1) the high prevalence of limb motor dysfunction in post-IS patients coupled with a low recovery rate; (2) DTI's distinct advantage in visualizing the occurrence of WD in CST associated with motor function. In examining the correlation between DTI and motor function recovery post-IS, multiple investigators use diverse perspectives, primarily involving two facets of CST and WM.

The extent of CST damage is intricately linked to the recovery of motor function. However, CST impairment has been assessed in different ways, with four main aspects: (1) Fiber number ratio of CST (FNr), both Bigourdan's team (3) (60) and Maraka's team (4) (61) used the FNr (affected side/healthy side) index to represent CST impairment and found it is significantly associated with outcomes in motor function after IS. (2) Kunimatsu et al. (5) (62) employed the DTI technique to illustrate the three-dimensional spatial relationship between the CST and infarcted lesions in patients with cerebral infarction. They discovered that muscle strength could be completely restored in patients with infarcted lesions adjacent to, but not encroaching upon, the CST. Conversely, in patients with infarcted lesions that encompassed the CST, muscle strength did not consistently improve, or the enhancement was not sustained over time. (3) CST lesion load (CST-LL), studies by Feng et al. (63) and Zhu et al. (64) calculated the CST-LL for each patient by superimposing the patient's injury maps with the probabilistic CST of control maps of healthy controls and found that this could be used to predict the outcome of the patient's motor function. (4) The DTI parameter FA, which was used in most studies as the FA of the CST or FA ratio (rFA = FA_ipsilesional_/FA_contralesional_), FA asymmetry (FA_asy_ = (FA_contralesional_ – FA_ipsilesional_)/(FA_contralesional_ + FA_ipsilesional_)) to predict patients' motor outcomes (24, 57, 65). A few investigators have used the parameters AD, MD, etc. Still, some studies (66) have shown that MD, AD, and RD-related indices are not as sensitive as FA-related indices in assessing the link between structural integrity and motor function of the CST. Moreover, numerous studies have varied about the timing of CST identification and the timing of motor prognostic assessment. Detection time ranged from 24 to 72 h post-onset in the study by Bigourdan et al. (60), from 1 to 4 days, 30 days, and 90 days post-onset in the study by Møller et al. (57), and from 5 to 30 days post-onset in the study by Kim et al. (67). The research conducted by Maraka et al. (61) demonstrated that DTI was a predictor of exercise results throughout several phases (acute, subacute, and chronic) following IS, with a more pronounced predictive capacity at the chronic stage. The research conducted by Thomalla et al. (19) indicated that DTI effectively predicted exercise outcomes three months post-IS; however, the study by Doughty et al. (68) revealed that the DTI parameter FA was an inadequate predictor of exercise outcomes at the same interval following IS. As evidenced by the aforementioned studies, there is still disagreement among researchers on CST detection techniques, detection durations, and prognostic times. This has important ramifications for how DTI is evaluated in clinical forecasting. Future study therefore intends to concentrate on determining the best prognostic time points and further investigating the best detection techniques and timings for CST.

For the WM aspect, numerous studies have demonstrated that utilizing DTI to assess the integrity of NAWM can forecast the transport function outcomes in patients with IS. However, the timeframe for these predictions has varied, including 72 h (40), 3–5 days post-admission (38), 90 days (41), and 1 year (42). Thus, continuing to figure out the optimal time points for predicting outcomes is also an avenue for further study.

Furthermore, in addition to the aforementioned predictions of IS outcomes by DTI techniques only, research indicates that the integration of DTI with supplementary techniques or scales may enhance the accuracy of motor outcome predictions. Stinear et al. (69) proposed that an algorithmic approach integrating transcranial magnetic stimulation of motor-evoked potentials and FA asymmetry forecasted the prognosis of patients' upper limb motor function, achieving a sensitivity of 73%, specificity of 88%, positive predictive value of 88%, and negative predictive value of 83%. Buch et al. (70) discovered that the integration of FMA and DTI at one week post-stroke effectively distinguished between patients exhibiting usual and atypical recovery patterns. It is noticeable that further investigation is necessary to determine if DTI technology should be utilized alone or in combination.

In general, numerous studies have determined that DTI technology is effective in predicting motor outcomes following IS and can serve as a biomarker for such predictions in IS patients. Nevertheless, the methodologies employed for testing, the specific objects of examination, and the forecasting of prognostic stages reflect divergent perspectives, resulting in a lack of general opinion. A consistent controlled comparison of the various concepts remains absent, and there is a deficiency of high-quality publications to validate the findings, demanding more investigation through large-sample, multi-controlled studies in the future.

## 4 Advantage and limitations

This study used a combination of three software or websites for bibliometric analysis to visualize and analyze different aspects (18), which may have a more comprehensive and objective character relative to other review articles. However, this study is further handicapped by the following shortcomings:

The included articles may have research bias: this study exclusively utilized articles from Web of Science databases, disregarding those from alternative databases such as Scopus. The limitations above may have caused an insufficient diversity of papers to be considered, ultimately resulting in study bias.

Limitations in the interpretation of bibliometric mapping: our analysis primarily concentrates on the network relationships among countries, institutions, or journals, along with emerging research trends. Consequently, we have not extensively interpreted the other nodes within the mapping; however, this does not imply that these nodes can be disregarded, resulting in specific limitations in the interpretative outcomes.

Lack of analysis of the various stages of IS: the bibliometric method is inadequate for analyzing each phase of a disease when considering the disease in its entirety. In the case of IS, a disease with clearly defined stages, this limitation hinders a comprehensive understanding of the research characteristics associated with each stage, thereby constraining the analysis of disease stages.

## 5 Conclusion

This study relies on bibliometric techniques to elucidate the knowledge map of DTI in IS from 2003 to 2024, analyzing the global trends in publication volume, the interconnections among publication sources, authors' contributions, and the evolution of research hotspots, aiming to provide researchers with a comprehensive overview of the field's research landscape and ideas. The findings indicate a year-on-year increase in global publication volume, suggesting heightened interest in this research domain from nations and research institutions, allowing researchers to explore this area further. Future research may concentrate on the following topics: (1) Whether the degree of CST damage and the structural integrity of the NAWM can independently serve as predictors of motor outcomes after IS; (2) Which detection method or parameter of the DTI technique is more effective as a biomarker of motor outcomes after IS; (3) How should the ideal detection time and the optimal outcome prediction time be chosen? (4) Whether the integration of the DTI technique with other methodologies or scales enhances the prediction of exercise outcomes in IS. Furthermore, it is imperative to bolster cross-regional collaboration among institutions, augment sample sizes and control groups, and amplify the study's focus and impact, thereby facilitating the improved application of DTI technology in IS and offering enhanced rehabilitation guidance for clinical IS patients.

## Data Availability

The original contributions presented in the study are included in the article/supplementary material, further inquiries can be directed to the corresponding authors.
